# Genome reannotation and gland-specific transcriptome analysis identify new effector candidates in Meloidogyne chitwoodi

**DOI:** 10.1371/journal.ppat.1013075

**Published:** 2025-11-07

**Authors:** Marcella Teixeira, Itsuhiro Ko, Sapinder Bali, Paulo Vieira, Thomas R. Maier, Thomas J. Baum, Cynthia Gleason

**Affiliations:** 1 Department of Plant Pathology, Washington State University, Pullman, Washington, United States of America; 2 Mycology and Nematology Genetic Diversity and Biology Laboratory, USDA Agricultural Research Service, Beltsville, Maryland, United States of America; 3 Department of Plant Pathology, Entomology and Microbiology, Iowa State University, Ames, Iowa, United States of America; University of California Davis, UNITED STATES OF AMERICA

## Abstract

The root-knot nematode (RKN) *Meloidogyne chitwoodi* is a threat for potato production in the western United States (U.S.), negatively impacting potato yield and product value*. Meloidogyne chitwoodi* produce proteins, called effectors, in their esophageal glands that are secreted during parasitism and play integral roles in plant-nematode interactions. Because the esophageal glands are the main effector secretory organs, we isolated juvenile gland cells and performed gland transcriptome analysis with our newly improved genome annotation. The gland-specific transcriptome data gave us an enrichment of gland-localized genes, which was validated by *in situ* hybridization. The gland transcriptome analysis led to the identification of 125 effector candidates. One of the effectors that was highly expressed in the pre-parasitic J2 gland tissue, referred to as McGland26, was further characterized. *Arabidopsis thaliana* expressing *McGland26* showed enhanced susceptibility to *M. chitwoodi*. However, the ectopic expression of *McGland26 in planta* did not suppress plant defenses, suggesting that this effector might be involved in processes other than interfering with plant immunity. Our data show that by using the gland transcriptome, a good quality genome annotation, and stringent selection criteria, we can increase the efficiency of effector identification, which can be used to develop more sustainable management tools.

## Authors summary

The root-knot nematode *Meloidogyne chitwoodi* is a major problem for potato farmers in the western U.S., reducing crop yield and quality. These nematodes produce special proteins, called effectors, in their esophageal glands, which help them infect plants. Since these glands are the main source of effectors, we isolated them from juvenile nematodes and analyzed their gene expression using our newly annotated *M. chitwoodi* genome. This helped us identify genes specific to the glands. We found 125 potential effector genes. One of them, called *McGland*26, was highly active before the nematode started feeding. When we introduced this gene into *Arabidopsis thaliana* plants, they became more susceptible to nematodes. However, expression of the effector didn’t seem to weaken the plant’s immune system. This suggests *McGland*26 is not suppressing plant defenses and has a different role in helping the nematode with successful infection. Overall, our approach helped us identify key effectors more efficiently, which could lead to better ways to manage nematode infestations in the future.

## Introduction

Root-knot nematodes (RKN, *Meloidogyne* spp.) cause substantial damage to crop plants, amounting to millions of dollars in agricultural losses each year [1]. *Meloidogyne chitwoodi* is a RKN that is endemic to the potato-producing regions of Oregon, Idaho, and Washington, where over half of the U.S. potatoes are grown [2,3]. This species is problematic for potato growers because the nematodes can infect both the roots and tubers. The tuber infections result in potatoes with a rough, bumpy appearance and brown spots under the skin, and there is little industry tolerance for such potato blemishes [4]. Moreover, *M. chitwoodi* is considered a quarantine pest in certain export markets for potatoes grown in the U.S., and their presence in a shipment bound for such a market could mean that the entire shipment is rejected [4]. There is currently no genetic resistance in commercially available potato cultivars in the U.S. [5]. To develop new strategies of *M. chitwoodi* management, we need information about how the nematodes are successful at parasitism, which calls for investigation into nematode parasitism genes, also known as effector genes.

Nematode effector genes encode proteins that facilitate successful infections, either by suppressing the plant immune responses and/or by altering the plant cell metabolism to form the nematode feeding site [6]. The expression of effector genes can occur at different life stages. Following embryogenesis and initial development in the egg stage, the RKN will hatch as a second-stage juvenile (J2). This is the motile infective life stage that must enter the plant root and migrate to the vasculature to establish its feeding site. The J2 releases several effector proteins, including cell wall-degrading enzymes, such as cellulases and polygalacturonases. Once inside the root, the J2 chooses between 6–10 plant cells to convert into its feeding sites called giant cells [7]. The plant cells that form into giant cells undergo synchronous waves of mitotic activity uncoupled from cytokinesis, and they are transcriptionally active, resulting in enlarged, multinucleate giant cells that the nematodes rely on for nutrition. After initial feeding, the J2 will quickly molt three more times into the adult stage. The sedentary adult female will feed on the giant cells and produce eggs in a gelatinous matrix on the surface of the root.

Because nematode parasitic success requires root penetration and giant cell formation, the effectors secreted during early stages of parasitism, in the pre-parasitic nematode J2 and during early feeding site formation (parasitic J2), are critical [8]. The pre-parasitic J2 faces the challenge of penetrating the roots and migrating towards the vascular cylinder. During this time, effectors must suppress any basal plant defenses, such as those inadvertently triggered by the pathogen associated molecular patterns (PAMPs), such as ascaroside #18 [9]. These basal plant defenses, known as PAMP-triggered immunity (PTI), are counteracted by nematode effectors that specifically inhibit PTI responses [8,10].

The identification of J2 effectors is not only interesting from the viewpoint of understanding nematode parasitism, but these effectors could also be targets for gene silencing as a possible future nematode control strategy. As a result, we have focused our current efforts on the pre-parasitic J2 transcriptomes. Previously, we studied the motile *M. chitwoodi* and sedentary parasitic J2 life-stages, and we reported that 2,248 genes were up-regulated in parasitic nematodes in potato roots at 8 days post-inoculation (dpi) compared to the motile, pre-parasitic J2 life stage [11]. Of these 2,248 genes, 269 contained a signal peptide (SP) sequence and no transmembrane (TM) domain(s). These two characteristics are key criteria often used in effector discovery pipelines. While bioinformatic predictions based on the presence of a signal peptide sequence and absence of transmembrane domains can help identify potential effectors, additional confirmation is typically needed. This is usually done through *in situ* hybridization (ISH) assays. The ISH assays are performed for each gene of interest to determine whether the transcript is localized to the secretory organ (e.g., esophageal gland cells). Most nematode effectors are found to be expressed in the esophageal glands and then secreted out of the nematode stylet into the plant [12]. Thus, the presence of a probe signal localized in the gland region of the nematode is indirect confirmation of secretion. Unfortunately, such assays are laborious, and in previous experiments, only about 20% of the transcriptionally upregulated genes in whole J2 transcriptome analysis that fit the traditional effector criteria (contain SP sequence and no TM domain) could also be confirmed to be gland localized by ISH [13]. As a result, gland transcriptomes may offer a more gland-targeted approach to effector discovery. Gland transcriptomes have been optimized for pinewood, cyst, root-knot and root-lesion nematodes, and have been useful in plant-parasitic nematode effectoromes characterization [14–19].

Here we have undertaken a gland transcriptome analysis of *M. chitwoodi* pre-parasitic J2s using a curated reannotated genome. This data has allowed us to identify effector candidates that are gland localized and relevant for early stages of parasitism. The gland transcriptome has shown that pre-parasitic J2s express known effectors, as well as novel, previously uncharacterized effectors. We selected a few effectors (both known and novel) and confirmed their gland localization by ISH, indicating that the gland library transcriptomes can confidently provide sets of effector candidates. By identifying and characterizing pioneer effectors through gland transcriptome analysis, we better understand nematode parasitism, which will ultimately facilitate developing effective management strategies for this economically damaging nematode species.

## Results

### Evidence-guided genome annotation improved the existing annotation of the *M. chitwoodi* genome

To better predict *M. chitwoodi* protein-coding genes from transcriptome analyses, we improved the *M. chitwoodi* genome annotation by reannotating the publicly-available genome assembly of *M. chitwoodi* (hereon referred to as version 1, V1) (S1 Fig) [1]. The reannotation was performed by using the *M. chitwoodi* RNA-seq datasets from Zhang *et al.,* 2021 [5] together with protein sequences from both closely and distantly related nematode species. To evaluate the quality of our reannotation, we performed Benchmarking Universal Single-Copy Orthologs (BUSCO) analysis to estimate the presence of conserved core orthologs in metazoa [2]. The reannotated *M. chitwoodi* genome (hereon referred to as version 2, V2) achieved a higher BUSCO score (71%) compared to V1 (48.7%), indicating improved genome annotation (Table 1).

**Table 1:** Genome annotation features for RKNs. The table shows a comparison between previous gene annotations for RKN and *M. chitwoodi* annotation version 2. BUSCO scores were calculated based on eukaryota_odb10 dataset [20].

Species	Genome pattern	Complete (%)	Single (%)	Duplicated (%)	Fragmented (%)	Missing(%)	Reference
*M. chitwoodi*	*AB*	71	67.2	3.8	3.8	25.3	[21]
*M. chitwoodi*	*AB*	48.7	47.7	1	7.4	43.8	[21]
*M. graminicola*	*AB*	69.7	63	6.7	5	25.3	[22]
*M. hapla*	*AB*	68.6	66.9	1.7	4	27.5	[23]
*M. arenaria*	*AAB*	73.9	2.6	71.3	4.1	22	[22]
*M. arenaria*	*AABB*	74.1	1.6	72.5	4.1	21.8	[22]
*M. arenaria*	*AABB*	73	8.6	64.4	4.5	22.5	[24]
*M. incognita*	*AAB*	72.1	10.2	61.9	4.5	23.4	[24]
*M. incognita*	*AAB*	73.7	3.7	70	3.7	22.6	[22]
*M. javanica*	*AABB*	72.6	8.1	64.6	4.3	23.1	[24]

**Fig 1:**
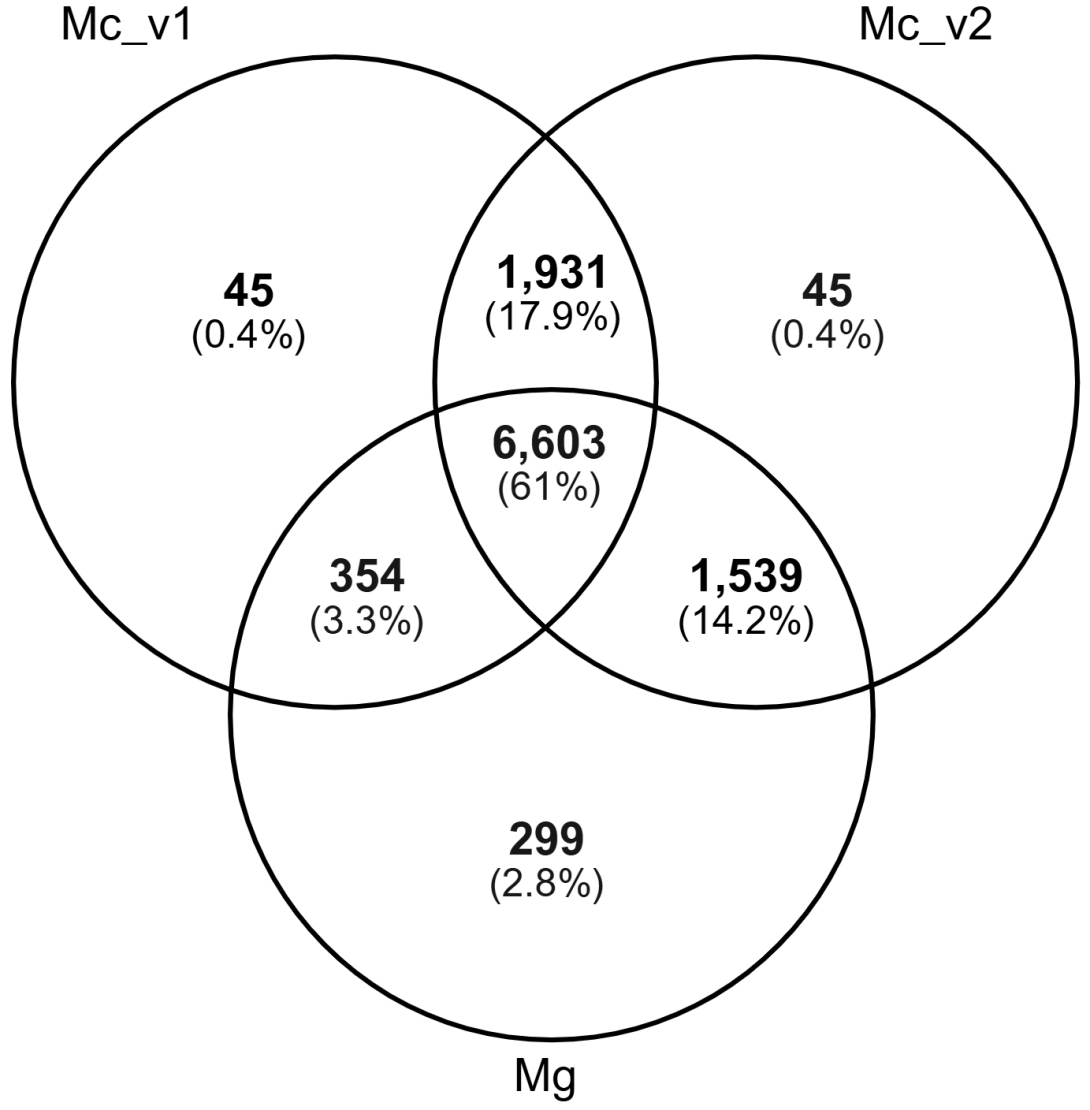
The newly re-annotated *M. chitwoodi* genome V2 shares more orthogroups with *M. graminicola* than with the V1 *M. chitwoodi* genome annotation. Venn diagram showing the shared gene families (orthogroups) between *M. chitwoodi* annotation V1*, M. chitwoodi* annotation V2, and the *M. graminicola* genome annotation.

To further evaluate V2 completeness and quality, a species-shared and a species-specific gene family (orthogroup) analysis was performed using Orthofinder [3], in which we compared the genome annotations of *M. chitwoodi* V1, V2, and its close relative *M. graminicola* [4,5]. This comparison was designed to test two hypotheses: Hypothesis 1)

the V1 and V2 annotations of *M. chitwoodi* have identical orthogroup profiles; and Hypothesis 2) because both annotations were derived from the same genome assembly, only a very small number of gene families should be unique to either V1 or V2, while being shared with *M. graminicola*. Among the 10,816 orthogroups identified across the three genome annotations, 8,534 (78.9%) were shared between the two versions of the *M. chitwoodi* annotations (V1 and V2). However, 1,248 genes (10.7% of all genes) in V1 lacked orthologs in any of the three annotations, which is a higher proportion than observed in V2 (4.1%). This finding does not support Hypothesis 1 and instead suggests that V1 likely contains artifact genes. Furthermore, 1,539 orthogroups (14.2%) were shared between *M. chitwoodi* V2 and *M. graminicola* but absent from V1 (Fig 1), contradicting Hypothesis 2. Collectively, these results indicate that the V2 annotation provides a more complete representation of gene families and therefore reflects a better annotation quality compared to V1.

### *M. chitwoodi* gland transcriptome analysis revealed putative effectors

The nematode esophageal gland cells are transcriptionally active during parasitism and are known to produce a large number of effectors [6]. To identify *M. chitwoodi* gland-specific effectors, total mRNA isolated from esophageal glands from pre-parasitic J2 of *M. chitwoodi* race 1 were sent for RNA sequencing [7]. The resulting gland transcriptomes generated between 18.3 to 43.3 million high quality raw reads in each of three bio-replicates. Kraken2 (v2.1.6, [8]) was performed on all gland transcriptomes against “core_nt” database. 34.07% of reads were unclassified and 44.52% of reads were classified as cellular organisms. Among these true reads, only 16.27% are from Metazoa (2.86% were assigned to Viridiplantae, 18.17% were assigned to Pseudomonadati). Around 0.7 to 6.8 million reads were mapped to the *M. chitwoodi* genome (S1 Table). Although the number of mapped reads appears relatively low, it is around the same percentage of mapped reads found in the *Pratylenchus* gland library [9] and may reflect: a large proportion of read mapping to contaminants (i.e., non-nematode) and/or the high stringency of the gland transcript analysis pipeline in mapping to the genome. Nevertheless, based on bioinformatic effector criteria (SP, but no TM), we identified 262 putative effectors from the gland transcriptome library generated for *M. chitwoodi* (S2 Table).

**Fig 2:**
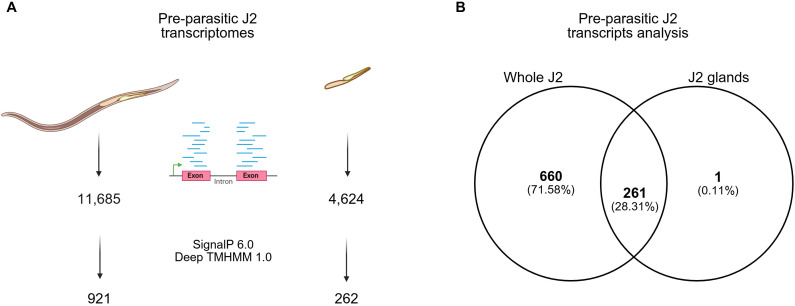
Transcriptome analysis summary. **A.** Comparison between the number of transcripts retrieved from whole J2s and their glands. **B.** Venn diagram showing number of overlapping putative effectors identified in pre-parasitic J2 and their glands. Created in BioRender. Teixeira, **M.** (2025) https://BioRender.com/f42i870.

Next, using the V2 genome annotation, we revisited the previously published J2 transcriptome data [5]. The new analysis resulted in 11,685 transcripts identified from the whole pre-parasitic J2 transcriptome, compared to the 4,624 transcripts identified from the gland transcriptome in all three biological replicates. Using the same bioinformatic criteria, 921 putative secreted proteins were retrieved from the whole J2 transcriptome (Fig 2). The comparison between whole J2 and the gland transcriptome revealed 261 genes in common between the two libraries (Fig 2 and S3 Table). Ten out of 261 genes that were common to both the whole J2 and the gland transcriptome showed sequence similarity to previously described RKN effectors (S4 Table). The normalized read counts of all 10 known effectors were assessed, and *McGland125*, a putative homolog of the effector MiXyl-1 [10] had the lowest read count from the gland transcriptome. Therefore, we have set the expression level of this gene as the minimum level of expression to be considered as an effector gene. Using this threshold, we narrowed our putative effectors

candidates to 125 genes (S5 Table). Out of the 125, 62 contained protein domains that are associated with nematode effectors, including those encoding calreticulins, glycoside hydrolases, C-type lectin-like proteins, and peptidases (S5 Table) [11].

**Fig 3:**
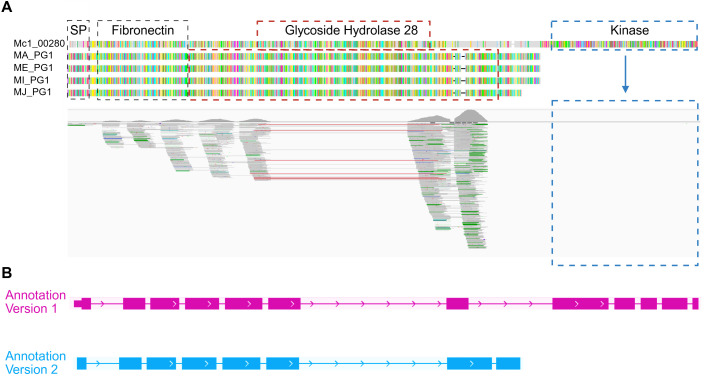
Genome reannotation improved the *M. chitwoodi* gene structure prediction. **A.** Protein alignments of polygalacturonases from *M. chitwoodi* (Mc1_00280), *M. arenaria* (MA_PG1), *M. enterolobii* (ME_PG1), *M. incognita* (MI_PG1) and *M. javanica* (MJ_PG1), showing that only *M. chitwoodi*’s V1 annotation predicts a protein with a kinase domain. **B.** Alignment of gland transcriptome reads, V1 (in pink) and V2 (in blue), validating the gene structure predicted by V2. Domains are highlighted in dashed boxes and domains that differ from the others in *Meloidogyne* spp. are in red and blue. Protein alignment was performed using Genious software and transcriptome alignment to the genome was performed using IGV. Created in BioRender. Teixeira, **M.** (2025) https://BioRender.com/o29t552.

The gland transcriptome data also gave us the opportunity to compare V1 and V2 genome annotations in the context of effector genes. We observed that several putative effectors were not accurately captured by the V1 genome annotation. For instance, the transcript sequence of *McGland75*, which was identified in the gland transcriptome, was used to query the V1 genome by a BLASTp search. The search identified *Mc1_00280*, which, based on sequence similarity and domains, is a possible homologue of MiPG1 (polygalacturonase 1) [12]. However, based on the V1 gene annotation, *Mc1_00280* gene structure was unusual; it encoded fibronectin and glycoside hydrolase family 28 domains, which are typical for polygalacturonases, and an additional kinase domain, atypical for polygalacturonases (Fig 3A). The V2 genome annotation predicts that *McGland75* encodes a protein with only fibronectin and glycoside hydrolase family 28 domains. Alignment of gland transcriptome reads for *McGland75* further verified that the V1 genome annotation was incorrect as there are no reads aligning to the kinase domain region (Fig 3B). This demonstrates that *McGland75* encodes a protein with typical polygalacturonase domains, and it demonstrates that the V2 genome annotation can better determine effector genes structures compared to V1.

### *M.* *chitwoodi* gland transcriptome helped identify gland-localized effectors

Interestingly, out of the 125 candidate effectors from the gland transcriptome analysis, 63 had no identified domain(s) based on either Interpro or NCBI Conserved domain database searches (S7 Table), nor were they sharing sequence homology to each other. Of the 63 genes analyzed, 42 showed moderate homology (>50% identity) to proteins from other *Meloidogyne* species, whereas the remaining 21 genes lacked homologs, suggesting they may be *M. chitwoodi*-specific candidate effectors (S6 Table). These 21 putative species-specific effectors were subsequently examined by comparing their transcript abundance between pre-parasitic and parasitic J2 stages [5]. Most of these genes (20) are highly expressed by the pre-parasitic J2 stage compared to the parasitic life stage (8 dpi in potato roots) (Table 2), showing they are more relevant for earlier stages of parasitism, likely before the establishment of feeding sites.

**Table 2:** Transcripts of putative *M. chitwoodi-*specific effectors, with unknown domains. Table shows transcripts per million (TPM) from pre-parasitic J2s and for parasitic nematodes at 8 dpi. Fold change was calculated relative to pre-parasitic J2 TPMs.

Gene id	J2	8dpi	Fold change
McGland18	13,678.04	0	-*
McGland71	1,952.42	0	-*
McGland26	1,629.16	0	-*
McGland100	128.56	0	-*
McGland37	85.95	0	-*
McGland94	4.48	0	-*
McGland31	66,013.78	5.35	12,341.31
McGland74	17,024.10	1.83	9,286.13
McGland119	145.56	0.86	168.96
McGland52	73.51	0.53	138.44
McGland17	23,066.48	253.04	91.16
McGland56	97.15	1.39	69.94
McGland93	26.28	0.8	32.7
McGland48	1,086.70	35.13	30.93
McGland53	119.9	5.52	21.73
McGland1	5,467.68	434.85	12.57
McGland88	43.42	4.56	9.51
McGland67	75.77	13.72	5.52
McGland19	1,089.95	218.25	4.99
McGland121	709.46	222.32	3.19
McGland101	56.67	92.5	0.61

*Transcripts not expressed at 8dpi.

**Fig 4:**
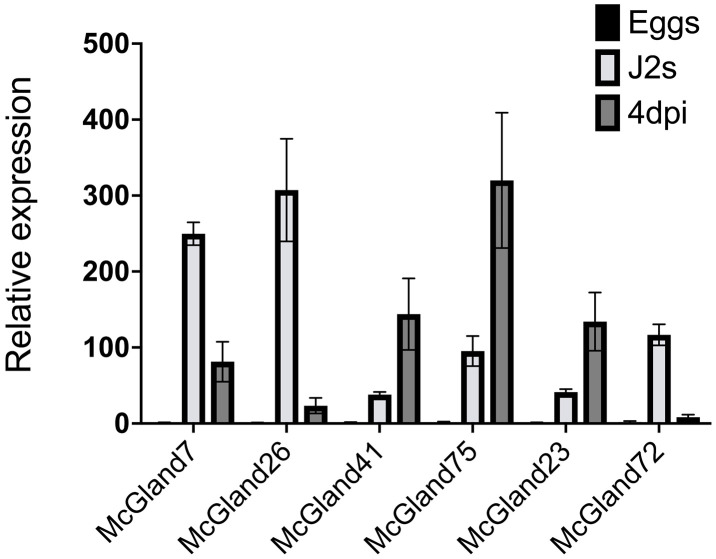
Relative expression of selected effector candidate genes at three nematode life stages. Relative gene expression was measured by qRT-PCR in eggs, pre-parasitic J2s and parasitic J2s at 4 days post-inoculation (dpi). Data show expression relative to egg stage (set to 1). **M. chitwoodi* ITS2* gene was used as internal reference gene. Data is presented as the mean fold change of two biological replicates + /- standard deviation (SD).

To confirm our *in-silico* identification of effectors from the gland transcriptome, we picked six genes to quantify their expression levels in three life stages (eggs, pre-parasiticJ2s and parasitic nematodes at 4 dpi) by performing qRT-PCR (Fig 4). The six genes are 1) a known *M. chitwoodi* effector - *McGland72*, which encodes a proteinase-inhibitor like protein [13]; 2) three putative effectors - *McGland41, McGland75*, and *McGland23,* which have sequence homology to the glycoside hydrolases *MiENG1* [14], *MiPG1* [12], and Mi-msp-1 [15], respectively; and 3) two unknown domain effectors - *McGland7* and *McGland26*. *McGland7* is a candidate effector with no predicted domains (Table 3) and putative homologs in other RKN species (S6 Table). *McGland26* is *M. chitwoodi-*specific based on BLAST analysis and shows one of the highest fold change differences in the comparison between the pre-parasitic J2s and the parasitic stage (8 dpi in potato roots) (Table 2). *McGland41*, *McGland75* and *McGland23* exhibited the highest level of expression in parasitic nematodes (4 dpi in potato) compared to eggs and pre-parasitic J2s (Fig 4). Transcript abundance of the two novel effectors, *McGland7* and *McGland26*, and the previously identified effector *McGland72* [13] were significantly higher in pre-parasitic J2s relative to the eggs and parasitic nematodes (4 dpi in potato).

**Table 3:** Transcripts chosen for further characterization. Domains were predicted using Interpro and NCBI Conseved Domain Database. Putative orthologs were obtained performing BLASTn searches on NCBI and WormBase parasite.

Gene id	Predicted domain	Putative orthologs
***McGland7***	Unknown	–
***McGland26***	Unknown	–
***McGland72***	Protease inhibitor-like	Mc11229 [25]
***McGland41***	Glycoside hydrolase family 5	MiENG1 [26]
***McGland75***	Glycoside hydrolase family 28	MiPG1 [27]
***McGland23***	VAP domain	Mi-msp1 [28]

**Fig 5:**
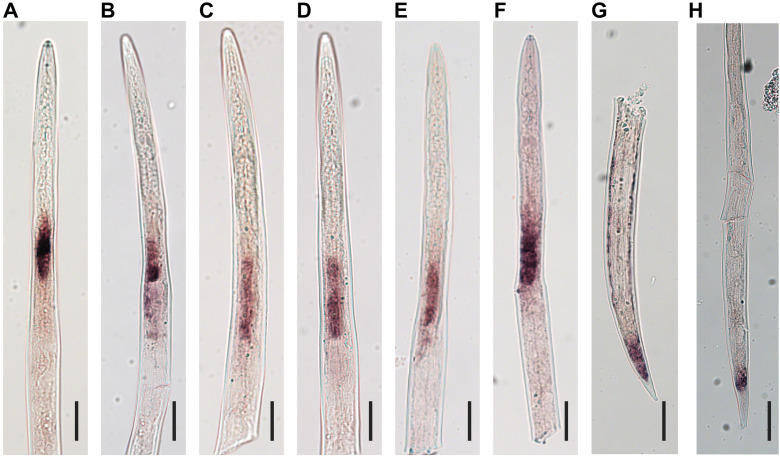
Representative photos of ISH of digoxigenin-labeled antisense probes for transcripts expressed in the J2s A-F, Gland localized effectors. **A**. McGland72 (described by Roze et al. 2018), **B.** McGland41 (putative glycosyl hydrolase with similarity to MiENG1), **C.** McGland75 (putative glycosyl hydrolase with similarity to MiPG1), **D.** McGland23 (effector with a cysteine-rich secretory proteins, antigen 5, and pathogenesis-related 1 proteins domain, with similarity to Mi-msp-1), **E.** McGland26 (*M. chitwoodi* specific), **F.** McGland7 (novel *M. chitwoodi* effector, with putative homologs in other RKN species). **G.** and **H.** Antisense probes for non-gland localized *M. chitwoodi* genes. Scale bars represent 25µm.

ISH was performed to confirm that all six genes localized to the esophageal gland region of the preparasitic J2 (Table 3 and Fig 5). To test the stringency of our effector discovery method, we tested two genes coding for putative secreted proteins (presence of SP and no TM) that did not pass our threshold of expression in gland tissue, McGene1 and McGene2. The ISH showed these transcripts were not localized to the glands, i.e., McGene1 localized to the hypodermis and tail and McGene2 localized to the tail region (Fig 5). Thus, among the tested genes, only the putative effectors that passed our gland- specific criteria were proven to be expressed in the glands, validating the gland transcriptome data and our effector discovery method.

### The novel effector McGland26 facilitates *M. chitwoodi* parasitism

*McGland26* stands out from other effectors because this *M. chitwoodi*-specific effector has no predicted functional domain and had one of the highest expression levels in the pre-parasitic J2 stage compared to other life stages, as observed in both our pre- parasitic versus parasitic J2 (8 dpi in potato roots) transcriptomes (Table 2) and in qRT-PCR analyses (Fig 4).

**Fig 6:**
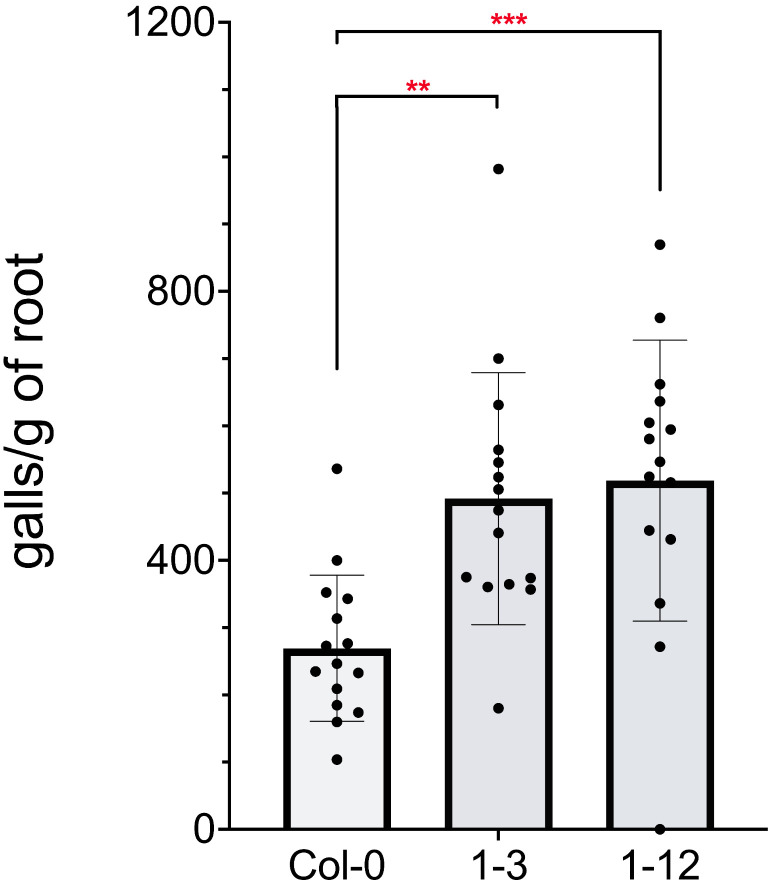
Expression of McGland26 in *A. thaliana* makes the plant more susceptible to *M*. *chitwoodi* infection. Two independent lines of *A. thaliana* expressing *McGland26* (1.3 and 1.12) and the wildtype control plants (Col-0) were inoculated with *M. chitwoodi* Race 1 J2s. Galls per plant and plant roots weights were assessed at 14 dpi. Data represents the mean number of galls per gram root + /- SD (One Way ANOVA with Dunnett multiple comparisons tests, **p-value ≤0.005, ***p-value ≤0.001). N = 15, per genotype. Experiment was repeated three times with similar results.

*McGland26* is a 279 bp coding sequence that encodes a 93 amino acid protein. The signal peptide sequence cleavage site was predicted to be between the amino acids 25 and 26. To characterize the role of *McGland26* in *M. chitwoodi* parasitism, two independent *A. thaliana* transgenic lines were generated that expressed *McGland26* without its signal peptide *(McGland26*^*Δsp*^*),* driven by the *CaMV35S* promoter. The transformation events in the two transgenic lines were first confirmed with genomic PCR and the expression of the *McGland26* in the two lines was measured by qRT-PCR (S2 Fig). The fresh shoot and root weight of the two transgenic lines showed no significant difference compared to wildtype plants (S2 Fig), suggesting that the expression of *McGland26*^*Δsp*^ has no effect on plant growth or development. *A. thaliana* lines expressing *McGland26*^*Δsp*^ were infected with *M. chitwoodi* and assessed at 15 dpi for root galling. Both transgenic lines exhibited more galling compared to the wildtype plants (Fig 6).

We hypothesized that the transgenic lines expressing *McGland*26^*Δsp*^ showed enhanced susceptibility to *M. chitwoodi* infection because the effector might be suppressing plant defense responses. We had previously identified a RKN effector that suppresses PTI by blocking defense-related callose deposition [16]. PTI responses, such as callose deposition and reactive oxygen species (ROS) production, can be quickly triggered in *A. thaliana* leaves by the bacterial defense molecule flg22 [17]. Therefore, we used flg22 to trigger these hallmark PTI responses in leaves of wildtype and transgenic *McGland*26^*Δsp*^ plants.

**Fig 7:**
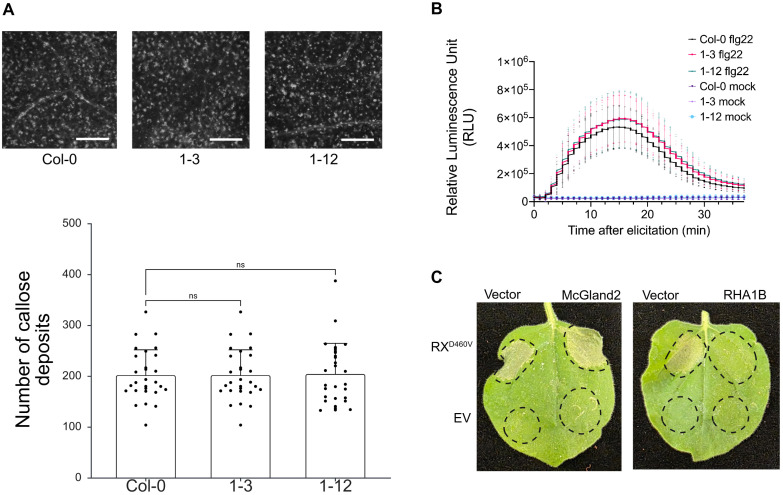
McGland26 does not suppress plant defense responses triggered by flg22. **A.** Quantification and representative images of aniline blue–stained callose deposits in *A. thaliana* leaves, comparing *McGland26*-expressing plants with wild-type plants, 24 hours after flg22 infiltration. Number of callose deposits in leaf areas were calculated using ImageJ software. The number of callose deposits in leaf areas was equivalent between transgenic *A. thaliana* plants expressing *McGland26* and wild-type plants after flg22 treatment. (n > 25 leaf discs). Bar = 200 µm. Experiment was repeated three times with similar results. **B.** Quantification of the ROS production after elicitation with flg22 in *A. thaliana* expressing *McGland26* and wild-type plants. Leaf discs were collected from the plants and subjected to flg22 induction, followed by measurement of relative luminescence by luminol assay. Graph shows relative luminescence units (RLUs) +/- SD of 18 leaf discs per treatment. **C.** Representative photos showing that McGland26 does not suppress HR mediated by the auto-active NB-LRR mutant *Rx1*^*D460V*^. *N. benthamiana* leaves were agro-infiltrated with a combination of *McGland26*, the potato cyst nematode *RHA1B* (positive control), and 35S::*GFP-HA* (empty vector-EV, negative control), either alone or in combination with the auto-active R gene mutant *Rx1*^*D460V*^. Photographs were taken 2 days after agroinfiltration. Experiment was repeated three times with similar results.

To determine if McGland26 would affect flg22-induced callose deposition in leaves, flg22 was infiltrated into the leaves of wildtype and *McGland26*^*Δsp*^ transgenic lines. Subsequently, flg22-induced callose deposits were detected using aniline blue staining*.* The leaves from both *McGland26*^*Δsp*^ transgenic lines showed similar numbers of callose deposits as the wildtype after flg22 treatment (Fig 7a).

Another hallmark of PTI after pathogen perception is the production of ROS [18]. A few RKN effectors have been shown to modulate the ROS, such as MjTTL5 [19], CATLe [29], and MjNEROSs [30]. Leaf discs from *A. thaliana* wildtype and *McGland26*^*Δsp*^ transgenic lines were treated with flg22, and ROS production was measured using the previously described luminol-based assay [27]. *In planta* expression of *McGland26*^*Δsp*^ did not significantly suppress flg22-induced ROS production compared with the control (Fig 7b).

Because we didn’t observe suppression of PTI responses by McGland26, we investigated the possibility that it might be involved in suppressing effector-triggered immunity (ETI). This represents a second level of plant defense, in which pathogen effectors are recognized, either directly or indirectly, by resistance (R) genes, triggering a strong defense response often seen as a hypersensitive response (HR) [31,26]. To test if the nematode effector McGland26 can suppress ETI, we used a known “autoactive” Rx1 resistance gene mutant called *RX1*^*D460V*^ [28]. When *RX1*^*D460V*^ was transiently expressed in *Nicotiana benthamiana* leaves, it triggered HR. Thus, this mutant construct serves as a constitutive inducer of ETI. When RX1^D460V^ was co-expressed with the potato

Cyst nematode effector RHA1B, cell death was suppressed, confirming the previous report that RHA1B suppresses this autoactive resistance response [25]. Co-expression of *McGland26*^*Δsp*^ and *RX1*^*D460V*^ in *N. benthamiana* did not suppress HR, suggesting that McGland26 does not have a role in supressing ETI (Fig 7c).

## Discussion

Proper prediction of protein-coding genes in nematode genomes is essential for effector identification and subsequent characterization. Among RKNs, *M. arenaria* has one of the best annotated genomes, with a BUSCO score of 74.1% [22]. In contrast, the *M. chitwoodi* V1 annotation (ASM1518303v1) has the lowest BUSCO score reported to date, at 48.7% (Table 1). In addition to the low BUSCO score, the V1 genome annotation had incorrect gene models, exemplified by the manual curation of an effector with homology to the well-characterized MiPG1 (Fig 3). These issues led us to reannotate the *M. chitwoodi* genome, resulting in genome annotation V2.

Genome re-annotation can benefit from both transcriptome and proteome data, which confer more robustness to annotation prediction [32]. Therefore, to improve the *M. chitwoodi* genome annotation, we combined our previously published J2 transcriptome and the current gland transcriptome datasets, with protein datasets from other plant-parasitic nematodes (PPNs), animal-parasitic nematodes, and free-living nematodes. The number of predicted genes in V2, 12,597, remains very similar to the V1 annotation (12,295). However, the BUSCO score increased for V2 to 71%, indicating that V2 has a better prediction of gene structure. The quality of this updated *M. chitwoodi* annotation (V2) was further validated by comparing the orthogroups between V1, V2, and the *M. graminicola* genome [4].

The previously reported whole pre-parasitic J2 transcriptome analysis indicated that there were 783 putative secreted effectors [5]. However, the transcriptome data represented transcripts from the entire nematode body, and the majority of known secreted effectors are produced in the nematode esophageal glands [33]. Therefore, focusing on genes expressed within the nematode glands is a more efficient way to identify putative effectors relevant for successful parasitism. For instance, gland transcriptomes generated for other nematode species, such as *Globodera rostochiensis*, *Pratylenchus penetrans, Radopholus similis, Bursaphelenchus xylophilus, M. incognita*, *Heterodera schachtii*, and *H. glycines* [6,9,34–37] revealed large sets of highly confident effector candidates. In the case of *M. chitwoodi*, 261 transcripts coding for secreted proteins were found in both the whole J2 and the gland transcriptomes. This suggests that, akin to other PPNs, the transcriptome of the gland library offers a more refined and targeted pool of potential effector candidates. By focusing on gland-specific putative effectors, it allows for a more precise identification of key molecular players, providing a higher level of specificity compared to broader approaches. It should be noted that not being gland specific does not preclude genes from being effectors as they may be secreted from nematode organs (e.g., amphids or hypodermis) other than the stylet.

To further refine the list of gland-specific effectors, we applied a conservative criterion, retaining only genes with expression levels higher than the known effector with the lowest expression profile within the gland libraries, resulting in a final set of 125 highly confident putative effector genes. These represent around 2.7% of the 4,624 total transcripts initially retrieved from our gland transcriptome. Other publications found that putative effectors represent 2.42% [6] and 7.5% in *M. incognita* glands [38], and 2% in *P. penetrans* gland transcriptomes [9]. These variations might reflect the different bioinformatic strategies deployed for effector discovery or nematode biology of these specific-species. For instance, our approach used known effectors in RKN to define a minimum level of effector gene expression. This stringent filtering step may exclude true effectors that have expression lower than this cutoff but allowed us to narrow our list of effectors to a more manageable number of candidates.

Among our final candidates, 49.6% encode proteins with known domains, many of which have been found in effectors from other RKN species. The remaining transcripts are novel proteins with unknown domains, from which 16.8% encode *M. chitwoodi* specific proteins and 33.6% encode proteins with similarity to other RKNs. Thus, our results show a small proportion of candidate genes encoding pioneer sequences, which suggests that most of the effectors were present before diverging from the last common ancestor.

To characterize novel effectors from *M. chitwoodi* involved in the early stages of parasitism, we analyzed their expression using our previously published transcriptome data from whole pre-parasitic and parasitic J2s [5]. Among the 21 *M. chitwoodi*-specific effectors, 20 were found to be highly expressed in pre-parasitic J2s compared to parasitic nematodes. Of these, McGland26 was selected for further study. When expressed in *A. thaliana*, *McGland26* increased the plant’s susceptibility to *M. chitwoodi*. While many RKN effectors are known to suppress plant defense responses, McGland26 did not suppress flg22-induced callose deposition or ROS accumulation, suggesting it may target other aspects of PTI or perform additional roles in facilitating nematode-plant interactions. Additionally, McGland26 did not suppress cell death triggered by the transient expression of an autoactive resistance gene, which indicates that it does not have a role in ETI suppression. Future studies will further explore the role of McGland26 in nematode parasitism and identify its interacting plant protein(s).

## Materials and methods

### Reannotation of *M. chitwoodi* genome

*M. chitwoodi* softmasked genome (ASM1518303v1) was obtained from Wormbase ParaSite database under Bioproject ID: PRJNA666745 [1]. The genome annotation was conducted at the high-performance computer cluster “Kamiak”, at Washington State University and a pre-built genome structural and functional annotation workflow [39] was adapted in this study. In brief, *M. chitwoodi* gland transcriptome generated from this study and previously generated *M. chitwoodi* J2 RNA-seq data [5] were processed using the transcript analysis workflow GEMmaker (v2.1.0) [40] using the Nextflow system [41]. STAR aligner [42] implemented in GEMmaker was used to map the transcriptomes to the *M. chitwoodi* race 1 softmasked genome [1]. The generated BAM (Binary Alignment Map) files were used as hint files for BRAKER1 [43], an RNA-seq based genome structural annotator. As suggested by Gabriel *et al.* 2021 [44], the annotation accuracy can be enhanced by running the protein homology-based genome annotator BRAKER2 with a large dataset of proteins from distantly related species to *M. chitwoodi* [45]. Therefore, the sequences of proteins from Invertebrate OrthoDB [20] were used in combination with protein sequences from the available genome annotations of 1) three RKN species (*M. javanica, M. arenaria, M. incognita*), 2) more distantly related PPN, *H. schachtii* (cyst nematode), 3) animal-parasitic nematodes *Trichinella spiralis, Loa loa, Brugia mallayi, Necator americanus*, and 4) free-living nematodes *Caenorhabditis remanei, C. elegans, and C. briggsae*. Next, TSEBRA [44] was used to combine the BRAKER1 and BRAKER2 results with the following configuration: “P 0.1, E 10, C 5, M 1, intron_support 0.75, stasto_support 1, e_1 0, e_2 0.5, e_3 25, e_4 10”. To further improve the annotation, EviAnn was used to annotate genes purely based on the same set of protein and RNA-seq data. Bedtool2 intersect [46] was used to combine the non-overlapped gene model from EviAnn and *M. chitwoodi* version 1 gene annotations [1] to the annotation file generated from the BRAKER pipeline (GFF file) and the merged annotation files were tidied using GenomeTools “gt-gff3 –tidy” function (v1.6.5) [47] and manually curated. The final structurally annotated *M. chitwoodi* genome assembly was fed into EnTAPnf [48] for gene functional annotation with gene ontology assignment. The completeness of functionally annotated genome assembly was assessed by testing the total protein sequence against metazoa_odb10 dataset using BUSCO [2]. The enrichment of gene expression in the gland transcriptome was visualized using Integrative Genomics Viewer (IGV) software (v. 2.10.3) [49]. The species-specific gene family (orthogroup) analysis was adapted from Zhang et al. (2022) [3]. Orthofinder [50] was used to assign each gene to a protein family.

### Transcriptomic analysis

Five to 10 esophageal glands were extracted from pre-parasitic second stage juveniles of *M. chitwoodi* race 1 and pooled to form one sample, as previously described [7]. Libraries were prepared using Takara Bio’s SMART-seq mRNA LP (634768) kit, which includes RNA isolation and library preparation. Three libraries were sequenced by Novogene, using Illumina Novaseq 6000 (PE 150 bp with 250M read depth).

The gland RNA-seq raw reads were trimmed for adaptors using Cutadapt (v2.10. [51,52]. With the parameters:”a AAGCAGTGGTATCAACGCAGAGTAC -a A -a T”. Salmon aligner [42,53] implemented in GEMmaker was then used to map the clean reads to all primary transcripts identified in the *M. chitwoodi* v2 genome annotation, generating transcript abundance estimates specific to expressed genes [54]. To make a comparison among different samples, transcript abundance was normalized into transcripts per million (TPM).

### Prediction of putative effectors

For possible effector gene identification from all transcripts, signal Peptides were predicted using SignalP6.0h (fast) [55,56] and transmembrane domains were predicted using DeepTMHMM1.0 (v.1.0.44) [57], both using default settings. Domain prediction was performed using Interpro [58] and NCBI’s conserved domain database [59] using amino acid sequences. To identify homolog of our candidate effector genes, Diamond (v2.1.8) [60] was used to search against: 1) Complete UniProtKB/Swiss-Prot data set (obtained from March 22^nd^, 2025) [61]; 2) NCBI BLAST non-redundant (nr) protein dataset (obtained from March 22^nd^, 2025)[59]; 3) RefSeq invertebrate protein dataset (obtained from September 11st, 2025) [62]; and 4) UniProtKB/TrEMBL all coding sequences CDS dataset (obtained from March 22^nd^, 2025) [63] respectively using the “very-sensitive” mode. Manual curation of putative effector gene structure was performed by visually aligning the RNA-seq reads from the gland transcriptome data to the exons of the predicted gene structure using Integrative Genomics Viewer (IGV) software v. 2.10.3) [49]. The names of putative effectors were numbered considering their expression level in the gland transcriptome.

### Cloning and transgenic lines generation

*McGland26* coding sequence, without its predicted signal peptide sequence (*McGland26*^*Δsp*^), was amplified from *M. chitwoodi* race 1 cDNA by PCR (S7 Table) and inserted into the Gateway donor vector pDONR207. It further was recombined into Gateway destination vector pB2GW7 to produce 35S::*McGland26*^*Δsp*^. After sequencing, the plasmid was used to transform *Agrobacterium tumefaciens* GV3101, which was used to transform *A. thaliana* Col-0 following the floral dip protocol of Clough and Bent (1998) [64]. Homozygous T3 seeds from two independent transgenic lines were collected from T2 lines after segregation analysis on BASTA-containing medium. Nematode gene expression in the transgenic plants was confirmed by qRT-PCR with *AtActin2* as a gene reference (S7 Table) [65].

### Nematode cultures

*M. chitwoodi* race 1 (initially provided by Dr Charles Brown from USDA-ARS, Prosser, WA) was multiplied on susceptible tomato *Solanum lycopersicon* cv. Rutgers under greenhouse conditions. Egg mass collection and J2 hatching was performed as previously described [5].

### RNA extraction and Real time PCR validation

Susceptible Russet Burbank plants were propagated in tissue culture for three weeks and then transferred to 500 ml cone-tainers filled with sand and subsequently maintained in growth chambers at 14 h:10 h light:dark cycle, 23 °C. Fourteen days after transplanting to sand, the plants were inoculated with 500 freshly hatched *M. chitw*oodi J2s. Samples were collected 4 days dpi and immediately frozen for RNA extraction.

For gene expression validation, three biological samples of eggs, pre parasitic J2s and galled potato root tissue at 4 dpi were collected. Total RNA was extracted using the Trizol method. Briefly, Trizol was added to ground sample and RNA was precipitated overnight using isopropanol. RNA was then purified using ethanol and resuspended in DEPC water. 1 µg of RNA was treated with DNA-free DNA Removal Kit (ThermoFisher) to remove residual DNA and ProtoScript II First Strand cDNA Synthesis Kit (New England Biolabs, USA) was used to prepare cDNA. Quantitative PCR was performed using SsoAdvanced Universal SYBR Green Supermix on a CFX96 Real-Time PCR Detection System (Bio-Rad, USA) and the primers listed in S7 Table. The qRT-PCR conditions were: 95C for 3 min, 40 cycles for 95C for 15 sec, 53C for 15sec and 72C for 20 sec, followed by a melting curve analysis from 65C to 95C with 0.5C increments at 5sec. Each experiment consisted of three technical replicates per sample.

Expression levels of genes of interest in nematode were normalized to the expression of Internal transcribed spacer 2 (ITS2) rRNA, a housekeeping gene from *M. chitwoodi* [5]. The relative expression levels of each target gene from pre parasitic J2s and 4 dpi tissue were calculated by comparing with those in eggs using the 2–ΔΔCt method [66].

### In situ hybridization (ISH)

Whole mount ISH were performed for *M. chitwoodi* J2 following the protocol of de Boer et al. 1998 [67]. Specific PCR primers were used to amplify the genes of interest from *M. chitwoodi* J2 cDNA (S7 Table). The resulting PCR products were then used as a template for generation of sense and antisense DIG-labeled probes using a DIG-nucleotide labeling kit (Roche). Hybridized probes within the nematode tissues were detected using an anti-DIG antibody conjugated to alkaline phosphatase and its substrate. Nematode segments were observed using a Nikon Eclipse 5*i* light microscope.

### Nematode infection assays

*A. thaliana* seeds were surface-sterilized and sown on Murashige & Skoog (Duchefa) agar plates (½MS salts, 1% sucrose, 0.8% agar, pH 6.4). Three-week-old seedlings were transferred to 500 ml sand-filled cone-tainers kept in growth chamber (14 h:10 h light:dark cycle, 23 °C). One week after transplanting to sand, the plants were inoculated with 750 *M. chitwoodi* eggs per plant. At 15 days post-inoculation, the plants were gently removed from the cone-tainers. Each root system was weighed, and the number of galls per plant was assessed. Nematode infection assays were repeated twice with similar results.

### Callose deposition and Reactive oxygen species (ROS) burst assays

For the callose deposition assay, leaves from 4-week-old *A. thaliana* plants were infiltrated with 1µM flg22. After 24h, leaves were punched using a cork borer and fixed in 95% ethanol overnight. Samples were then rehydrated with a serial incubation with 50% ethanol for 30 minutes, followed by two 30 min incubations with a phosphate-buffered saline, stained with 0.8% aniline blue for 1h and visualized under an AxioObserver A1 inverted microscope using a DAPI filter. Callose deposition was quantified with ImageJ [68]. At least 25 leaf punches were collected from four plants per genotype using a 4 mm cork borer, all images were taken at 10X magnification, and the experiment was repeated twice.

For the ROS burst assay, leaves from 4-week-old *A. thaliana* plants were punched using a cork borer and placed into a 96 well plate with 200 µl of double distilled water. The plate was closed and placed in dark overnight. The next morning, the ddH_2_O was replaced with 150 µl of fresh ddH_2_O and incubated for 1h. For the assay, the water was replaced with 100 μl of the oxidative burst assay solution containing 20 mg of HRP per liter (essentially salt free; Sigma), 2 mM luminol derivative (L-012; Wako, Richmond, VA, U.S.A.), and the bacterial elicitor flg22 added at a final concentration of 1 μM. The time-dependent increase in chemiluminescence was monitored using a GloMax Navigator luminometer (Promega, Madison, WI, U.S.A.) [69].

### Agrobacterium-mediated transient protein expression and western blotting

After cloning McGland26 without its predicted signal peptide sequence (*McGland26*^*Δsp*^), it was recombined into Gateway destination vector pK7FWG2 to produce 35S::**McGland39*Δ*sp with** a GFP tag. After sequencing, the plasmid was used to transform *A. tumefaciens* GV3101, which was used to syringe-infiltrate *N. benthamiana* leaves. As control, a modified pB2GW7 vector with cloned GFP-HA was used, generating 35S::*GFP-HA*. The construct 35S::*HA-RHA1B*^*Δsp*^ and the autoactive resistance protein RX^D460V^ was provided Dr. Fanming Xiao [25]. The concentration of agrobacterial inoculum varied from OD_600_ = 0.5 to 0.75. After 48 hours, Agrobacterium-infected *N. benthamiana* leaves were photographed, and samples were collected and snap frozen with liquid nitrogen.

For Western Blotting assay, the collected samples were ground to a fine powder and resuspended with 1 ml of protein extraction buffer (50 mM Tris-Hcl pH 8.0, 150 mM NaCl, 1mM EDTA, 5mM DTT, 2% NP40, 1x plant protease and phosphatase inhibitor cocktail – Thermo Scientific, Rockford, USA) and centrifuged at 15,000 rpm/4°C for 15 minutes. Protein samples were separated on SDS-PAGE gel, transferred onto PVDF membrane and probed with either anti-HA (Invitrogen, catalog #26183-HRP) or anti-GFP (Santa Cruz Biotechnology, catalog #SC-9996HRP) conjugated antibodies. Protein signal was detected with SuperSignal West Femto Maximum Sensitivity Substrate (Thermo Scientific, Rockford, USA, catalog #34095).

### Statistics

All statistical tests were performed using GraphPad Prism version 8.00 for Windows (GraphPad Software, La Jolla California, USA). One Way ANOVA with Dunnett multiple comparisons tests performed for nematode infection and callose deposits assay. All experiments were repeated at least three times.

## Supporting information

S1 Fig A schematic workflow of the genome annotation pipelines used in this study. Teixeira, M. (2025) https://BioRender.com/f18v715.
(TIF)

S2 Fig Evaluation of two independent lines expressing *McGland26* (lines 1.3 and 1.12). **A.** transgene expression from each line. **B.** Root weight of the two lines and Col-0 at 14 dpi. Data show mean weight of the roots + /-SD. (Mann Whitney test, error bars represent SD, ns: not significant). N = 15. The experiment was repeated three times with similar results.
(TIF)

S3 Fig Growth and development phenotype of *McGland26* transgenic lines 1.3 and 1.12. **A.** Representative photo of 2-week-old seedlings grown on MS media. **B**. Representative photos of the rosettes of the wildtype and transgenic plants after 1 month in the soil. C. Root lengths of 2-week-old seedlings. Data show mean lengths of the roots + /-SD. (Mann Whitney test, error bars represent SD, ns: not significant). N = 15. The experiment was repeated three times with similar results.
(TIF)

S4 Fig Mapped reads supporting McGland26 annotation. Representative mapped reads of McGland26 from the gland transcriptome aligned with annotations version 1 and 2. Version 1 annotation predicts 2 overlapping genes in McGland26 genomic region. Version 2 annotation predicts a smaller gene that matches the reads obtained from the gland transcriptome. Read mapping performed using IGV.
(TIF)

S5 Fig Western blot of infiltrated *N. bethaniama* leaves. The expression of the effectors *McGland26* and *RHA1B* in *N. benthamiana* leaves was confirmed by western blot (WB) using anti-GFP or anti-HA antibodies. (C, D) experiments were repeated three times with similar results.
(TIF)

S1 Table Summary of Gland transcriptome sequencing and alignment data. (XLSX)

S2 Table Gland transcripts with SP and no TM domain. (XLSX)

S3 Table Common transcripts between glands and J2. (XLSX)

S4 Table Putative homologs of known effectors. (XLSX)

S5 Table Putative Mc1 effectors after using homologs as cut-off criteria. (XLSX)

S6 Table Transcripts for which no domain was predicted using Interpro and NCBI Conserved Domain Database. (XLSX)

S7 Table Number of effectors encoding proteins with known domains. (XLSX)

S8 Table Primers used in this paper. (XLSX)
