# Supplementary figures and images for: Genome reannotation and gland-specific transcriptome analysis identify new effector candidates in Meloidogyne chitwoodi
Source: PLoS Pathog. 2025 Nov 7;21(11):e1013075. doi: 10.1371/journal.ppat.1013075 (PMC12614787; doi:10.1371/journal.ppat.1013075)

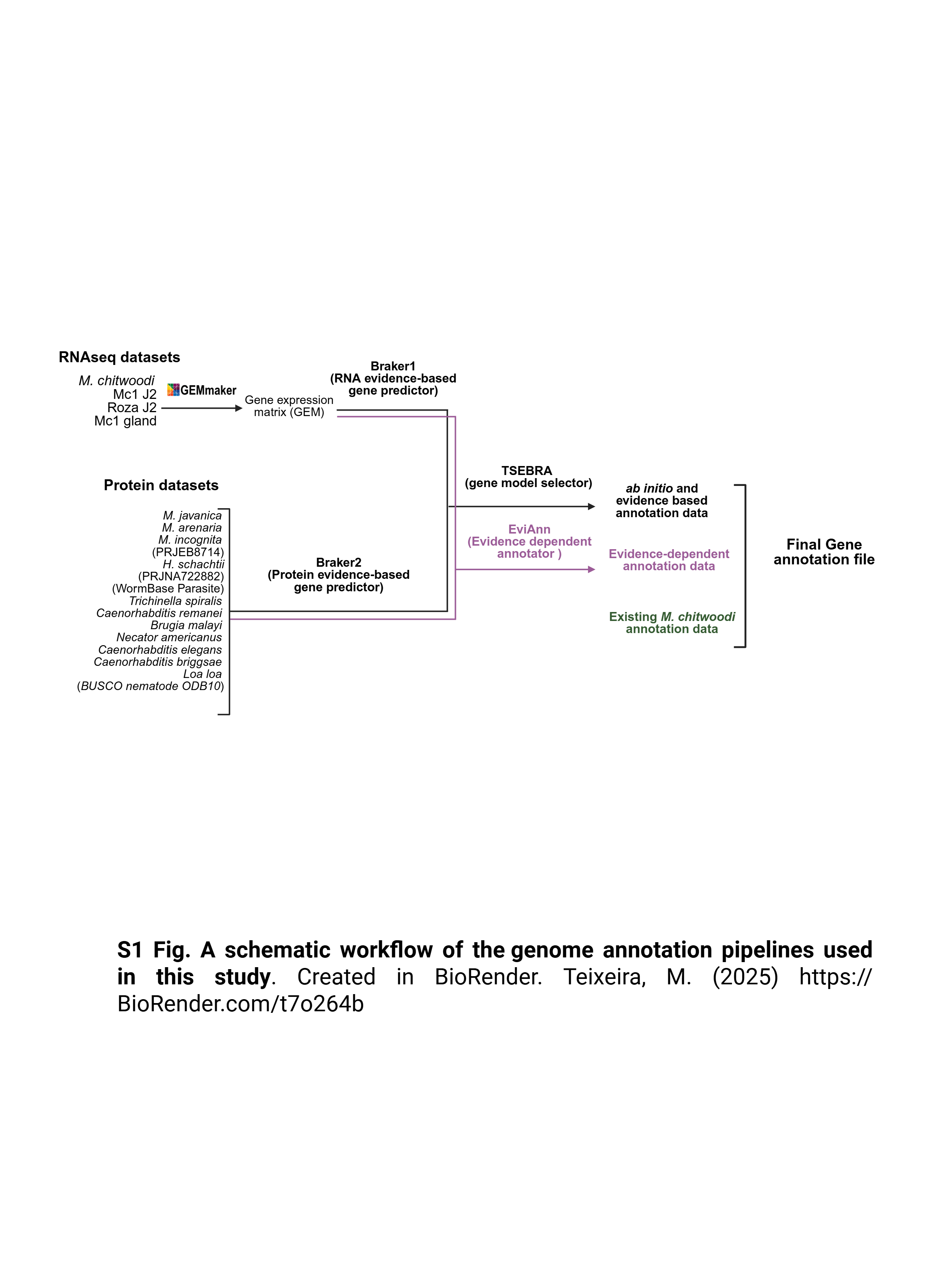

Supplement: S1 Fig — Teixeira, M. (2025) https://BioRender.com/f18v715. (TIF) [file ppat.1013075.s001.tif]

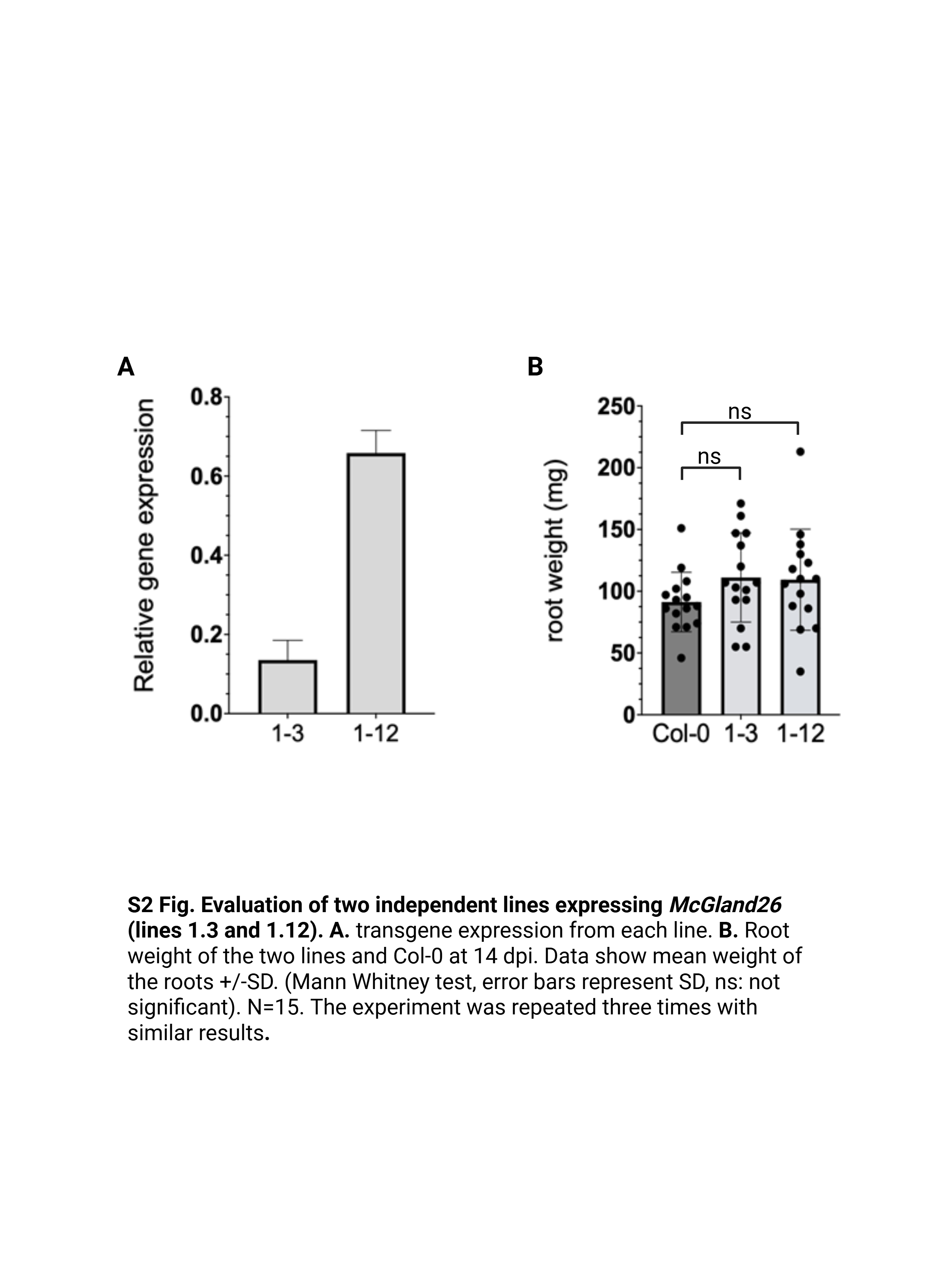

Supplement: S2 Fig — A. transgene expression from each line. B. Root weight of the two lines and Col-0 at 14 dpi. Data show mean weight of the roots + /-SD. (Mann Whitney test, error bars represent SD, ns: not significant). N = 15. The experiment was repeated three times with similar results. (TIF) [file ppat.1013075.s002.tif]

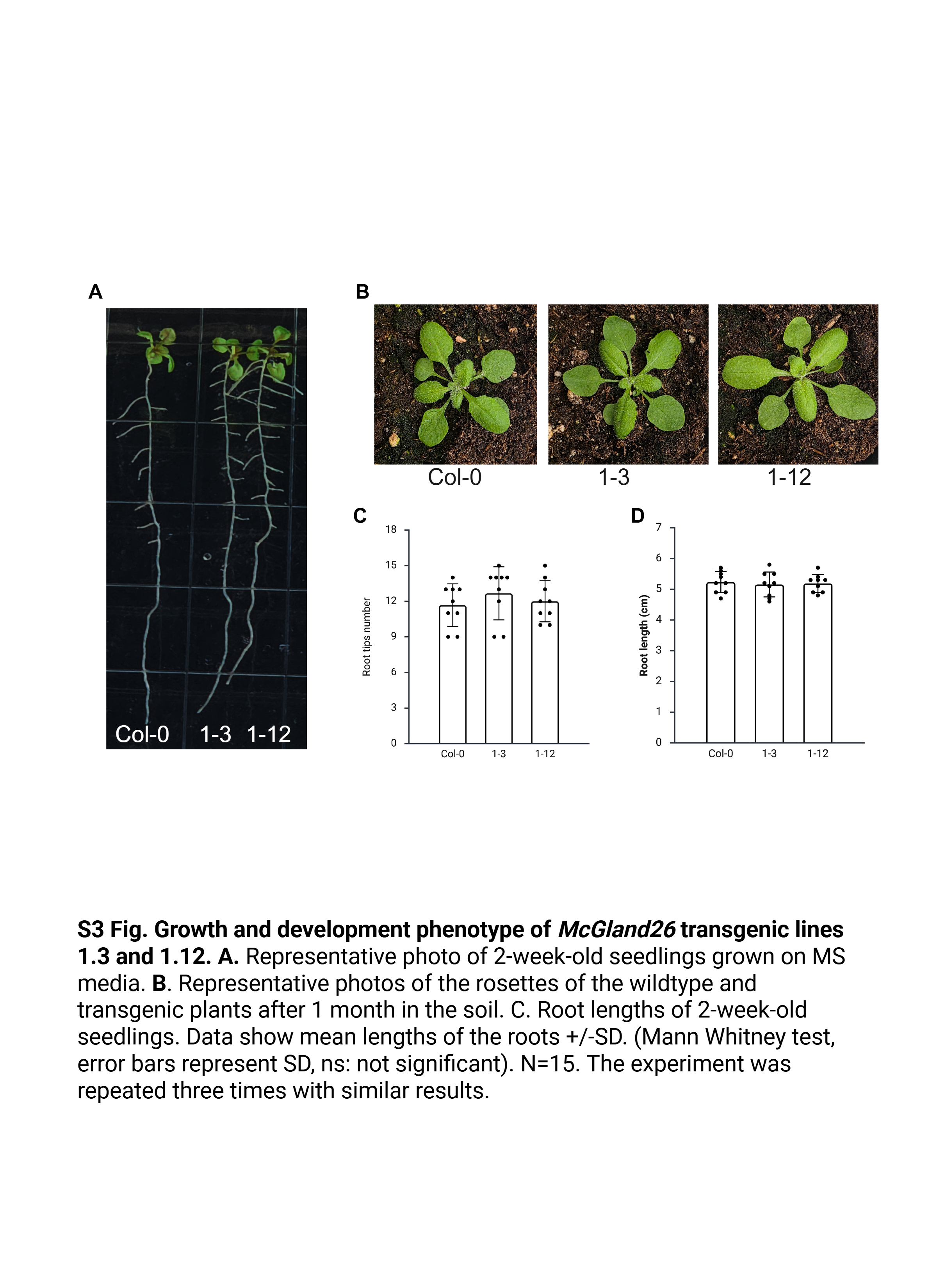

Supplement: S3 Fig — A. Representative photo of 2-week-old seedlings grown on MS media. B. Representative photos of the rosettes of the wildtype and transgenic plants after 1 month in the soil. C. Root lengths of 2-week-old seedlings. Data show mean lengths of the roots + /-SD. (Mann Whitney test, error bars represent SD, ns: not significant). N = 15. The experiment was repeated three times with similar results. (TIF) [file ppat.1013075.s003.tif]

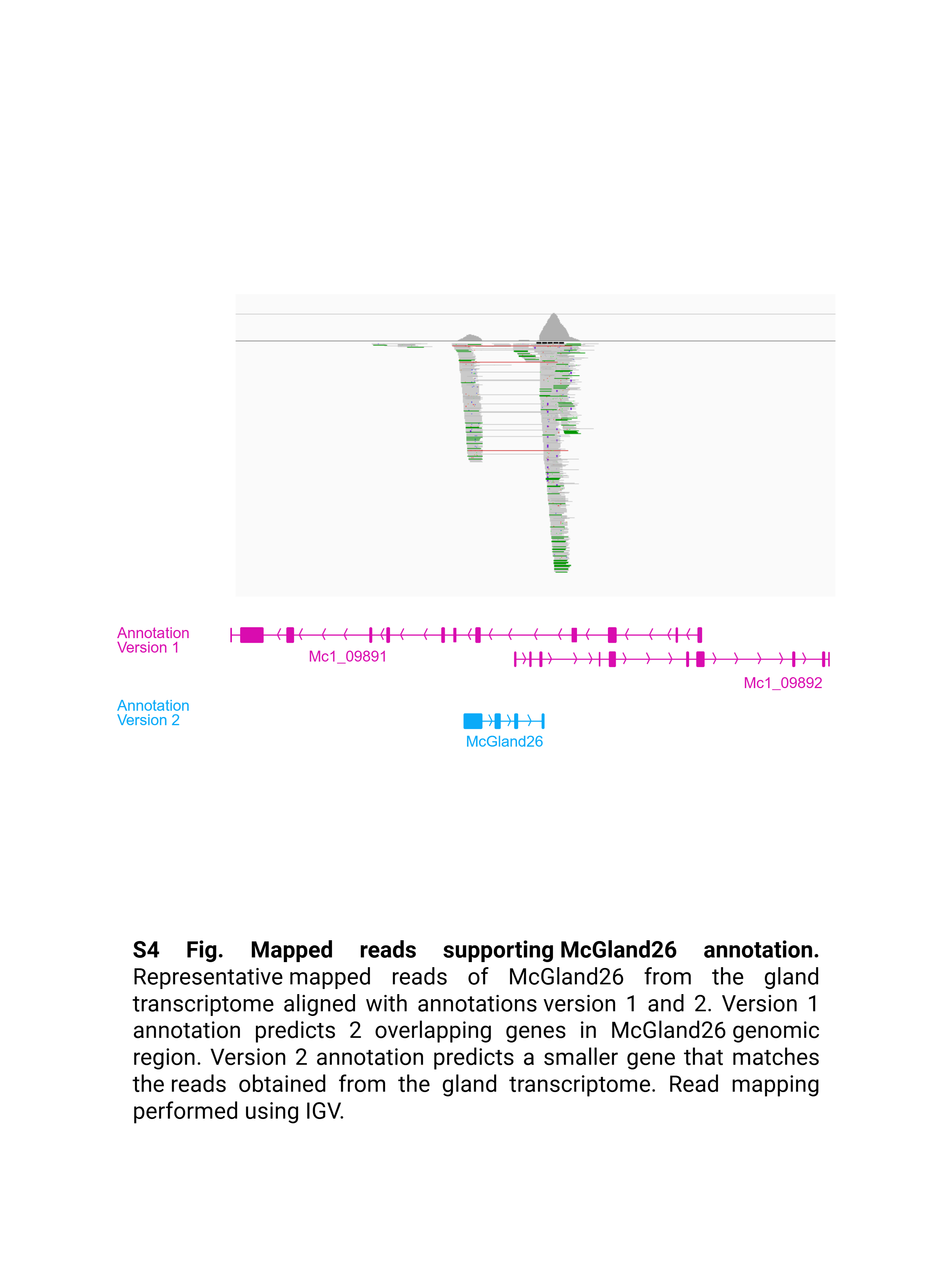

Supplement: S4 Fig — Representative mapped reads of McGland26 from the gland transcriptome aligned with annotations version 1 and 2. Version 1 annotation predicts 2 overlapping genes in McGland26 genomic region. Version 2 annotation predicts a smaller gene that matches the reads obtained from the gland transcriptome. Read mapping performed using IGV. (TIF) [file ppat.1013075.s004.tif]

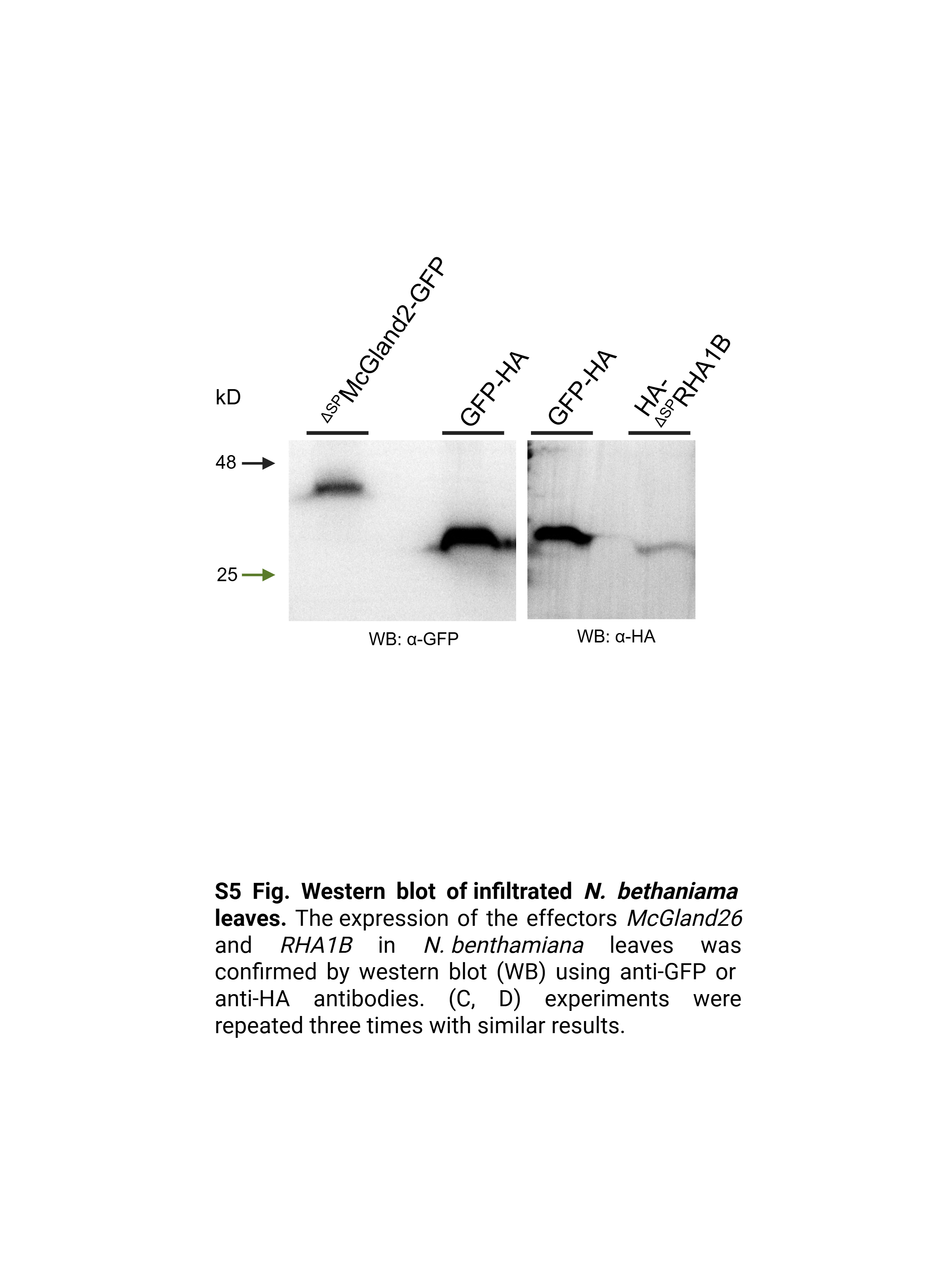

Supplement: S5 Fig — The expression of the effectors McGland26 and RHA1B in N. benthamiana leaves was confirmed by western blot (WB) using anti-GFP or anti-HA antibodies. (C, D) experiments were repeated three times with similar results. (TIF) [file ppat.1013075.s005.tif]
